# Effect of Polyglucosamine on Weight Loss and Metabolic Parameters in Overweight and Obesity: A Systematic Review and Meta-Analysis

**DOI:** 10.3390/nu12082365

**Published:** 2020-08-07

**Authors:** Simone Perna, Sana N. M. Basharat, Khawla F. Ali, Abdulla Eid, Clara Gasparri, Vittoria Infantino, Milena A. Faliva, Maurizio Naso, Roberta Cazzola, Benvenuto Cestaro, Mariangela Rondanelli

**Affiliations:** 1 Department of Biology, College of Science, University of Bahrain, Sakhir Campus P.O. Box 32038, Bahrain; sana.nazmb@gmail.com; 2 Department of Medicine, Royal College of Surgeons in Ireland-Medical University of Bahrain, Adliya P.O. Box 15503, Bahrain; kali@rcsi-mub.com; 3 Department of Mathematics, College of Science, University of Bahrain, Sakhir Campus P.O. Box 32038, Bahrain; aeid@uob.edu.bh; 4 Endocrinology and Nutrition Unit, Azienda di Servizi alla Persona ‘‘Istituto Santa Margherita’’, University of Pavia, 27100 Pavia, Italy; clara.gasparri01@universitadipavia.it (C.G.); milena.faliva@gmail.com (M.A.F.); mau.na.mn@gmail.com (M.N.); 5 Department of Public Health, Experimental and Forensic Medicine, University of Pavia, 27100 Pavia, Italy; viriainfantino@hotmail.it (V.I.); mariangela.rondanelli@unipv.it (M.R.); 6 Department of Biomedical and Clinical Sciences, “L. Sacco Hospital”, School of Clinical Nutrition, Faculty of Medicine and Surgery, University of Milano, 20157 Milano, Italy; roberta.cazzola@unimi.it (R.C.); benvenuto.cestaro@unimi.it (B.C.); 7 IRCCS Mondino Foundation, 27100 Pavia, Italy

**Keywords:** polyglucosamine, obesity, overweight, weight loss, waist circumference

## Abstract

The use of dietary supplements for weight loss has gained significant momentum. Polyglucosamine, a chitosan derivative, is a dietary supplement increasingly used for weight loss. In this meta-analysis, we systematically summarized and quantified the key findings of four randomized, placebo-controlled clinical trials examining the effects of polyglucosamine supplementation and caloric restriction, and physical activity on body weight, body mass index (BMI), and waist circumference in subjects with overweight and obesity. The control group was set with a physical activity from 6–7 MET-h/week activity and up to 21 MET-h/week activity with caloric restriction. Compliance in the latter trials was reported via a follow-up questionnaire with the individual participants. The analysis included 399 subjects followed for a period ranging from 12 weeks to one year. Subjects’ age ranged from 21–75 years, BMI from 26–45 kg/m^2^, and all were white European or Caucasian in ethnicity. The meta-analyzed mean differences for random effects showed that polyglucosamine supplementation improves weight loss by −1.78 kg [−2.78, −0.79], BMI by −1.52 kg/m^2^ [−3.58, 0.54], and improves waist circumference reduction by −1.45 cm [−2.77, −0.12]. In conclusion, the use of polyglucosamine supplementation in conjunction with lifestyle behavioral therapies can be effective for weight reduction. Further studies are needed to examine the long-term effects of polyglucosamine supplementation on weight loss and other metabolic parameters

## 1. Introduction

Overweight and obesity are amongst the most significant global health burdens and leading causes of morbidity and mortality worldwide. Excess weight, particularly obesity, is a major risk factor for development of type 2 diabetes mellitus, hypertension, hyperlipidemia, cardiovascular disease, and cancer [1].

With an ever-increasing burden of overweight and obesity, healthcare providers are strongly advocating for implementation of anti-obesity behavioral therapies, such as increased physical activity and dietary programs. However, with the strong biological internal defenses that resist weight loss, the simple advice of “eat better and exercise more” cannot be expected to produce meaningful and long-lasting weight reductions [2]. Therefore, the use of weight loss-aiding tools, such as pharmacological and supplemental agents, has become essential for any weight loss and/or maintenance intervention [2].

Recent guidelines by the European Society of Endocrinology and The Obesity Society recommend that diet, exercise, and behavioral modification be included in all obesity management approaches for body mass index (BMI) ≥ 25 kg/m^2^, in addition to other treatments such as pharmacotherapy (BMI ≥ 27 kg/m^2^ with comorbidity or BMI over 30 kg/m^2^) and bariatric surgery (BMI ≥ 35 kg/m^2^ with comorbidity or BMI over 40 kg/m^2^). In particular, the guidelines state that drugs may amplify adherence to behavior change and may improve physical functioning as increased physical activity is easier in those who cannot exercise initially [3,4]. 

The use of dietary supplements for weight loss has gained significant momentum in the past few years [5]. Nevertheless, the initial use of supplements for weight loss dates back to many decades ago. In the mid-1900s, with the increase in rates of overweight and obesity, several “diet pills” emerged on the market. These included supplements containing thyroid hormone extracts, diuretics, laxatives, and stimulants such as amphetamines [6]. However, with the increased incidence of adverse drug reactions, such as iatrogenic hyperthyroidism, electrolyte disturbances, and increase in cardiovascular events, the use of such supplements was deemed unsafe and inappropriate. The drug rimonabant, a diet pill, was withdrawn from the market in RIO-Europe, RIO-North America, and RIO-Lipids, due to its psychiatric effects (mainly depression) which occurred in 6−7% of rimonabant-treated individuals, which is an absolute increase of 2−5% over placebo [7]. Additionally, sibutramine, a sympathomimetic and appetite suppressant was also withdrawn from the market in the past decade due to concerns over increased cardiovascular events and stroke [8]. Currently, only a small number of FDA-approved drugs for long-term weight loss exist. Such drugs include liraglutide 3.0 mg, bupropion/naltrexone, orlistat, and phentermine/topiramate. 

There was a need for dietary supplement to aid with weight loss along with nutritional and exercise intervention. The dietary supplement, used frequently off-label for weight reduction, is polyglucosamine: a chitosan 80 derivative. Polyglucosamine is a low-molecular-weight chitosan acquired through marine sources and obtained after deacetylation of chitin with deacetylation degree (DD) of >70% [9,10]. Oral polyglucosamine acts by decreasing the bioavailability of enteral dietary fats via binding to fat molecules within the lumen of the gastrointestinal tract [11,12]. Polyglucosamine is a positively charged molecule that binds with high affinity to the negatively charged enteral fat molecules, thereby forming large-sized lipid-chitosan emulsions that are weakly digested [11,12]. The latter process eventually restricts the absorption of dietary fats into the blood stream, hence contributing to decreased energy intake and weight loss [11,12]. The hindrance of fat absorption may prevent the absorption of essential fatty acids and fat-soluble vitamins such as vitamins A, E, D, and K. Although little published evidence supports this with polyglucosamine, this may be extrapolated from the evidence based on the use of orlistat, a lipase-inhibitor, FDA-approved for medical weight loss [13]. Orlistat is known to hinder the digestion of fat and therefore its absorption, leading long-term to deficiencies in fat-soluble vitamins [13]. Therefore, monitoring and supplementation of such vitamins with polyglucosamine use may be clinically indicated [5]. There are not any specific guidelines or recommendations with regard to the prescription of polyglucosamine for weight reduction. A recent meta-analysis with only two preliminary studies by Cornelli et al. recommended the use of the administered dosage of 2 g/day (2 × 2500 mg tabs) to be taken before or during main meals for reducing weight by 5% [4]. However, further research is needed to verify the dosage and effectiveness of polyglucosamine in weight control.

Several clinical trials have investigated the effects of polyglucosamines on weight loss in subjects with overweight and obesity [9,11,12,14,15,16]. A few of these studies demonstrated significant reductions in body weight, whereas others were inconclusive. The heterogeneity in the supplementation programs likely contributed to the variability in weight loss outcomes. Additionally, the non-uniformity of concomitant lifestyle interventions added to the supplementation programs may have also played a role in the different outcomes [5,9]. 

With this meta-analysis, we aimed to systematically examine the effects of polyglucosamine on body weight, body mass index (BMI), and waist circumference in subjects with overweight and obesity. We aim to summarize and quantify the key findings of randomized, placebo-controlled, clinical trials conducted on polyglucosamine use for weight loss. 

## 2. Materials and Methods 

### 2.1. Search Strategy

English-written articles were identified by searching Google Scholar, WOS, PubMed, and Scopus databases. The search strategy was based on the following search terms: polyglucosamine and/or chitosan or hyperglycemia or cholesterol and/or dietary supplementation (MeSH terms). The search strings were then combed to link with subsequent words: weight-loss, obesity, overweight, dietary fibers, clinical trials. 

### 2.2. Study Selection

For this particular meta-analysis, a specific set of guidelines abbreviated as PRISMA (Preferred Reporting Items for Systematic Reviews and Meta-Analysis) was used [17]. This is an efficient approach that is evidence-based and allows effective reporting of systemic reviews of different research studies. The research was constructed around the following modules for better and more thorough evaluation: 1. participants, 2. interventions, 3. comparators, 4. outcomes, and 5. study design (PICOS).

The search was not restricted to specific languages, years of publication, or types of studies. In addition, reference lists of retrieved articles were searched manually for potential additional studies. The last search was carried out on 13 February, 2020. 

### 2.3. Data Extraction

All predefined outcome variables of included studies were extracted by two authors (S.P. and S.B.). Any disagreements during the selection process were resolved by a discussion with a third author (K.A.). The critical appraisal was carried out independently by two reviewers (S.P. and S.B.). In case of missing concordance, a third reviewer was included (K.A.).

A set of data was collected from each study, which comprised of the name of the first author, year of publication, setting and design of the study, eligibility criteria, number of subjects enrolled, subjects’ ethnicity or country of origin, sex and age of subjects, duration of intervention, and final outcome of the study. A meta-analysis for combined approximation for all the gathered data was then executed. 

### 2.4. Participants

Adult participants aged 18 years and above, with overweight or obesity, defined as BMI ≥ 25 kg/m^2^ were included in the analysis. No restrictions were placed on participant sex, ethnicity, presence of medical comorbidities, or location of study. 

### 2.5. Intervention and Control Group

Randomized, placebo-controlled and/or with other supplement, clinical trials that investigated the effects of polyglucosamine on body weight, waist circumference, and BMI were selected. Such studies included any double-blind placebo-controlled clinical trial that investigated the effects of either short- or long-term polyglucosamine supplementation, in tablet or capsule form, and those coupled with secondary interventions such as dietary behaviors and physical activity. 

In this meta-analysis, selected studies included the same setting and received similar lifestyle coaching/changes in the controlled arm such as with the intervention arm. The control group was set with a physical activity from 21 to 6–7 MET-h/week activity plus caloric restriction. Compliance in the latter trials was reported via follow-up questionnaire with the individual participants.

### 2.6. Outcomes

Outcomes obtained in studies from each arm (control and intervention) included body weight, BMI, and waist circumference measurements at baseline and at follow-up. The changes in these outcomes were then compared between the groups.

### 2.7. Inclusion and Exclusion Criteria

All studies conducted prior to 2008 were excluded. Only randomized, placebo-controlled, clinical trials were included. Subjects under the age of 18 years or with BMI < 25 kg/m^2^ were excluded. Only English-language articles were included. A separate review was conducted on studies examining the effects of polyglucosamine on body weight and/or metabolic parameters in animals. In addition, we considered all studies that lasted from 1 to 12 months.

### 2.8. Risk of Bias in Individual Studies

Cochrane Collaboration Software was used to assess the risk of bias [18]. The Risk of Bias Tool, as well as the generation of allocation sequence, allocation concealment, blinding of data, and completion of data and reporting were all used to assess the biasness of studies. Each of these factors was classified with low, high, or unclear risks of bias. Studies with low risk of bias in none or one item only were classified as poor, whereas those with low risk of bias in at least three items were classified as good. Studies with two items with low risk of bias were considered fair.

## 3. Results

**Figure 1:**
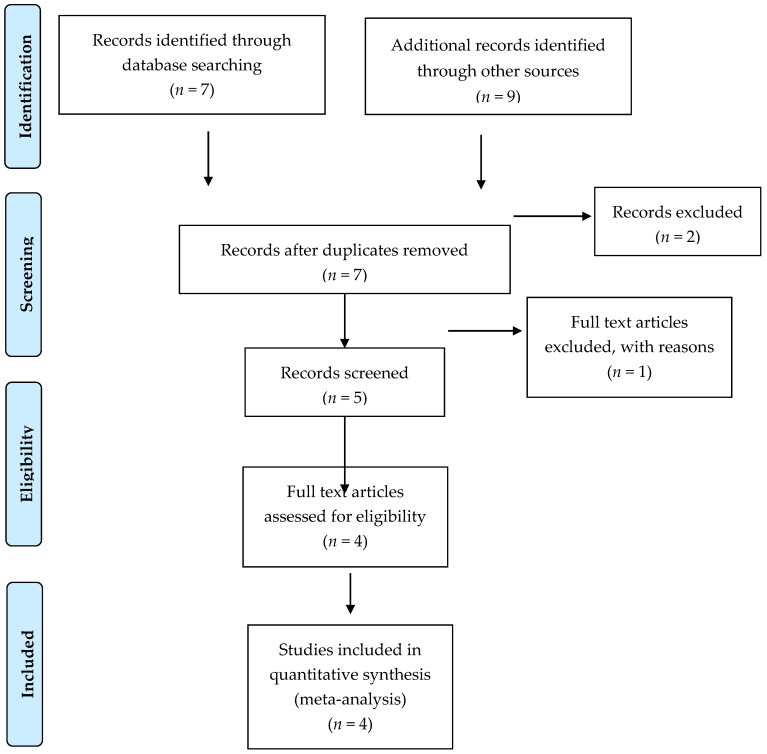
Flowchart.

The screening of literature for meta-analysis review resulted in a total of seven articles, of which only four met the inclusion and exclusion criteria of study (Figure 1) [12,14,15,16].

Table 1 summarizes the characteristics of the four selected clinical trials assessing the efficacy of polyglucosamine in weight loss. A total of 399 subjects, from both sexes, were included in the studies. Subjects’ age ranged between 21–75 years, BMIs were 26–45 kg/m^2^, and all were white European or Caucasian in ethnicity. 

In addition to polyglucosamine supplementation, participants in three studies were instructed to increase their MET-h/week and follow a calorie-restricted diet. In two studies, subjects were required to consume a protein- and fat/oil-rich diet. The polyglucosamine dosages in the studies ranged from 800 mg/day to 850 mg twice daily (Table 1). The primary outcome assessed to determine the efficacy of polyglucosamine supplementation was changes in body weight; BMI and waist and hip circumference were classified as secondary outcomes. The treatment duration lasted from 12 weeks to 12 months.

### 3.1. Meta-Analyzed Data

**Figure 2:**
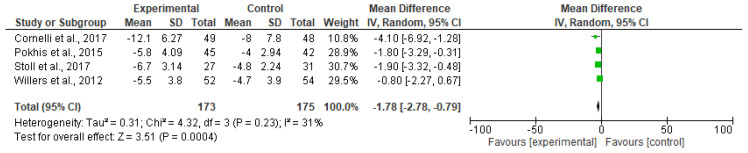
Forest plot of comparison: effect of polyglucosamine on body weight, versus placebo.

Polyglucosamine supplementation decreased body weight in all four studies. The mean placebo-subtracted weight loss across all studies was −1.78 kg (CI95% −2.78, −0.79 kg) (Figure 2). 

**Figure 3:**
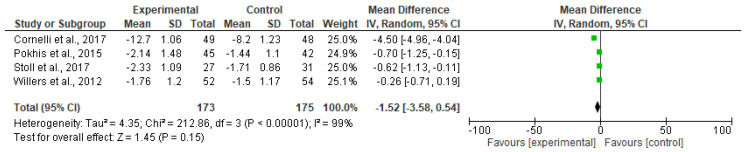
Forest plot of comparison: effect of polyglucosamine on BMI versus placebo.

All four studies showed reductions in BMI, one study showed a loss as high as −1.5 kg/m^2^ placebo-subtracted BMI [15]. The mean placebo-subtracted weight loss across all four trials was −1.52 kg/m^2^ (CI95% −3.58, 0.54 kg/m^2^; Figure 3). 

**Figure 4:**
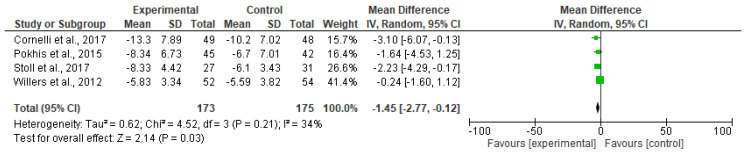
Forest plot of comparison: effect of polyglucosamine on waist circumference versus placebo.

Reductions in waist circumference were observed throughout, with mean placebo-subtracted waist circumference reductions of −1.45 cm (CI95% −2.77, −0.12 cm; Figure 4).

### 3.2. Risk of Bias

No publication bias was found for any of the outcomes, as determined via a funnel plot inspection and Begg’s and Egger’s tests (Table 2).

## 4. Discussion

This meta-analysis encompassed four randomized, placebo-controlled, clinical trials with nearly 173 subjects receiving polyglucosamine supplementation for weight loss, for a duration ranging from 12 weeks to one year [12,14,15,16]. Although many chitosan derivatives are currently being studied for weight loss, polyglucosamines remain the most efficacious [5]. Compared to other more complex and higher molecular weight chitosans, polyglucosamines’ low molecular weight, combined with their linear structure, allows for higher fat binding within the intestines [19]. 

Our results showed that polyglucosamine supplementation when combined with lifestyle interventions can have positive effects on body weight. Weight loss reported in the polyglucosamine arms ranged from 5.5 kg in one study [16] and up to 12.1 kg in another [15]. Weight loss of this magnitude is known to have significant benefits on health and well-being and carries marked improvements for many medical comorbidities. The benefits of the 5–10 kg weight reduction have been shown in several landmark clinical trials, such as the Diabetes Prevention Program (DPP) trial [20] and the Look AHEAD study (Action for Health in Diabetes) [21,22]. The latter trials showed that with only 5–10 kg reduction in body weight, significant clinical improvements can be seen in glycemic control, blood pressure, lipid profile, fatty liver disease, sleep apnea, physical function, and overall quality of life [20,21,22]. 

Polyglucosamine supplementation when combined with lifestyle interventions can also result in significant reductions in waist circumference, ranging from 5.8 to 13.3 cm [15,16]. Waist circumference is a vital health sign, and often a better predictor of disease risk than weight or BMI alone. A high waist circumference is known to be associated with elevated risk of diabetes mellitus, cardiovascular disease, renal disease, and metabolic syndrome. Thus, reductions in the magnitude seen in our analysis are key for reducing the risk of chronic disease occurrence and/or disease progression [23]. 

The likely benefits of polyglucosamines supplementation extend beyond their weight loss benefits to improving hyperlipidemic and hyperglycemic states. As polyglucosamines act primarily by inhibiting intestinal fat absorption, their use can lead to reductions in serum total cholesterol and triglyceride levels, as previously shown [11]. Additionally, polyglucosamine was shown to improve glycemic measures, mainly the hemoglobin A1c levels [16]. The improvements seen in blood glucose may be directly linked to weight loss. However, recent evidence suggests polyglucosamines can indirectly increase feces excretion of glucose, thereby improving glycemia independent of weight loss [11]. 

Additionally, as nutritional and physical activities are known to bring about meaningful weight loss estimated at 3.2–4.3 kg in a few studies [24,25], we only included studies with a placebo-controlled arm to exclude any non-pharmacological effects on weight loss. The average placebo-subtracted weight loss attributed to polyglucosamine intervention was −1.77 kg, which remains clinically important. The main limitation of this meta-analysis was the wide range of treatment duration (from 12 weeks to 12 months). This long duration could be a possible bias that has not been accounted for in the random effect analysis. 

Finally, despite several studies demonstrating the efficacy of polyglucosamines in combination with dietary interventions for weight loss [9,11,12,14,15,16,26,27,28], the effects of polyglucosamines alone have not been thoroughly investigated. A study by Pittler et al. [29] examining the effects of an oral chitosan supplement without dietary interventions found no significant weight reductions. Additionally, no studies have yet examined the dose–response relationship between polyglucosamine and weight loss [30]. 

The results of this meta-analysis showed a weight loss −1.78 kg [−2.78, −0.79]; the result related to another important meta-analysis on orlistat showed a mean weight difference between treatment and control groups of −2.10 kg (95% CI: −2.3 to −1.8 kg,), which are within the same lines [31]. 

## 5. Conclusions

The use of polyglucosamine supplementation, in combination with behavioral interventions, is effective at reducing weight, BMI, and waist circumference. Further studies are needed to examine the effects of polyglucosamine on metabolic parameters such as lipid profile, liver function profiles, glucose levels, and inflammatory markers. Additionally, the use of polyglucosamine in specific disease states, such as mixed hyperlipidemia, type 2 diabetes mellitus, and fatty liver disease ought to be investigated. 

## Figures and Tables

**Table 1:** Characteristics of polyglucosamine studies in humans.

First Author/Year of Publication	Subjects, Sex (Polyglucosamine vs. Placebo)	Population (Age, BMI)	Treatment Duration	Polyglucosamine Dosage	Control Group	Outcomes
Pokhis et al., 2015 [14]	Total: 115 (36 M, 79 F) (45 vs. 42)	21–75 years, BMI of >26 and <45 kg/m^2^	26 weeks	850 mg PG tablets twice daily + 6–7 MET-h/week activity + caloric restriction	6–7 MET-h/week activity + caloric restriction	Weight, BMI, and waist circumference
Cornelli et al., 2017 [15]	Total: 100 (50 M, 50 F) (49 vs. 48)	25–65 years, BMI of >30 and <35 kg/m^2^	12 months	Two 400 mg PG tablets twice daily + caloric restriction + 8 MET-h/week	Caloric restriction + 8 MET-h/week	Weight, BMI, waist circumference
Willers et al., 2012 [16]	Total: 120 (61 M, 59 F) (52 vs. 54)	30–60 years, BMI of 28–35 kg/m^2^	12 weeks	Two 400 mg PG tablets/day + Protein-rich formula diet	High protein-rich formula diet	Weight, BMI, waist and hip circumference, waist to hip ratio, blood glucose, and lipid parameters
Stoll et al., 2017 [12]	Total: 64 (28M, 36 F) (27 vs. 31)	21–70 years, BMI of >28 and <45 kg/m^2^	12 weeks	Two PG tablets twice/day	One orlistat capsule three times/day	Weight, BMI, waist circumference

F: female; M: male; PG: Polyglucosamine. MET-h: Metabolic Equivalent Task Hours/week (Physical activity). BMI: body mass index.

**Table 2:** Bias for studies included in the meta-analysis according to the Cochrane Risk of Bias Tool ^a^.

Study, Year	Random-Sequence Generation	Allocation Concealment	Participant-Personnel Blinding	Outcome-Assessment Blinding	Incomplete Outcome Data	Selective Reporting	Other Bias
Cornelli et al., 2017 [15]	Low	Unclear	Low	Unclear	Low	Low	Low
Pokhis et al., 2015 [14]	Low	Unclear	Low	Unclear	Low	Low	Low
Stoll et al., 2017 [12]	Low	Unclear	Low	Unclear	Low	Low	Low
Willers et al., 2012 [16]	Low	Unclear	Low	Unclear	Low	Low	Low

^a^ Bias designations by study criteria are indicated by seven domains with categories including low risk if negative aspects of the study design were not likely to influence the study findings, high risk if the study design was likely to influence the study findings, or unclear risk if high or low risk could not be assigned because of a lack of evidence.
